# Raising medical students’ awareness for the interdependence between oral health and systemic diseases - evaluation of a problem-based learning intervention: an exploratory pilot study

**DOI:** 10.1186/s12875-026-03323-4

**Published:** 2026-04-24

**Authors:** Katharina Pöschel, Anne-Kathrin Geier, Thomas Frese, Gerhard Schmalz, Anne Marie Schrimpf, Markus Bleckwenn, Dirk Ziebolz, Tobias Deutsch

**Affiliations:** 1 Institute of General Practice, Faculty of Medicine, Leipzig University, Philipp-Rosenthal-Straße 55, Leipzig, 04103 Germany; 2 Institute of General Practice and Family Medicine, Martin-Luther-University Halle-Wittenberg, Halle (Saale), Germany; 3 Department of Conservative Dentistry and Periodontology, Brandenburg Medical School (Theodor Fontane), Brandenburg/Havel, Germany

**Keywords:** Undergraduate medical education, Dentistry, Periodontitis, Surveys and Questionnaires, Germany

## Abstract

**Background:**

There are several bi-directional linkages between oral and systemic inflammatory diseases, such as diabetes and cardiovascular disease. Knowledge of basic mutual principles and close cooperation between dentists and general practitioners are therefore necessary to ensure optimal treatment of affected patients. Currently, oral health plays a subordinate role in German undergraduate medical curricula. To raise awareness of the importance of interprofessional collaboration with dentists and enhance students’ knowledge about oral health, a new learning session on oral health was integrated into the problem-based learning (PBL) course for 5th year undergraduate medical students in Leipzig.

**Methods:**

Pre-post online surveys were conducted, including items on students’ attitudes and perceptions regarding the importance of dentistry for their future medical work, and the need for cooperation with dentists. Self-assessed confidence on performing oral examinations and identifying patients at risk for dental complications was complemented by a multiple-choice test on periodontitis. The course format (PBL) and its integration into the curriculum were also assessed. Students from a neighboring university served as the comparison group. Both qualitative and quantitative data were collected. SPSS was used to perform descriptive statistics, and free text answers were analyzed using qualitative content analysis based on Mayring.

**Results:**

The response rate was 35.9% with 125 of 348 student participants completing both pre- and post-intervention surveys. The comparison group response rate was 86.6% with 188 of 217 invited participating. Participants showed a high interest in oral topics and great awareness both before and after the intervention. After the learning session, students felt more confident in performing oral examinations and identifying patients at risk for oral complications. Knowledge increased significantly, albeit on a low level. PBL was regarded as suitable for the integration of oral content, but students called for a stronger theoretical underpinning.

**Conclusions:**

This study provides indications that new interdisciplinary content in a PBL course for 5th year undergraduate medical students could be suitable for raising awareness for dental-medical interdependences and enhancing the interest in oral health topics in medical studies. The study also highlighted the need to close theoretical gaps related to oral health earlier in the curriculum, which students were open to. Based on validated instruments, this pilot approach should be further investigated to verify and scale the results.

**Supplementary Information:**

The online version contains supplementary material available at 10.1186/s12875-026-03323-4.

## Introduction

There are numerous connections between oral and systemic diseases. It is well established that inflammatory processes in the oral cavity, especially periodontitis, are strongly and bi-directionally linked with systemic inflammatory diseases such as type 2 diabetes and cardiovascular disease (CVD) [1–5]. Not only are patients with diabetes more prone to develop periodontitis, but periodontitis is also associated with a higher risk of CVD such as myocardial infarction, heart failure, and stroke [1, 3].

Consequently, improving the prevention and treatment of the respective conditions requires mutual professional knowledge and the exchange of medical information between physicians, namely general practitioners (GPs) and dentists [6–9]. Despite the importance of interdisciplinary cooperation between dentists and GPs, there continue to be significant shortcomings in daily practice in Germany [10–13]. This appears to be due to a lack of interest in cooperation, especially on the part of the GPs, as well as a lack of trust in each other’s specialized knowledge, and professional insecurities [10–12]. The lack of integration between these professions begins early, in undergraduate education. In Germany, as in many other countries, dental health plays a subordinate role in medical studies, and the lack of dental teaching content in undergraduate medical studies has frequently been criticized [12, 14–17]. There is an international consensus that knowledge and skills regarding oral health and its influence on systemic diseases should be included in the training of medical students to improve overall patient health, and subsequently improve and promote dental-medical collaboration [14, 15, 18]. Furthermore, dentistry and medicine are taught separately at university with little overlap. Although some basic courses are attended together in the first two years of study (e.g., biology, anatomy, chemistry), there is no explicit interprofessional exchange between medical and dental students in German medical education [12].

To fill gaps in medical students’ knowledge on oral health, raise their awareness for important interrelations between oral health and systemic diseases, and, thus, facilitate interdisciplinary cooperation, we integrated dental topics into the medical curriculum at the Leipzig University. The new teaching content was embedded in a problem-based learning (PBL) course in the field of geriatrics. This course is mandatory for all students in the 5th study year and is appropriate in terms of content, as older patients are particularly affected by systemic inflammatory conditions [19]. PBL is as an internationally recognized and well-established interactive teaching format. Compared to traditional learning, it has positive effects particularly on students’ communication and social skills, is fun for students, and achieves positive long-term effects on medical competencies, despite not being superior with regard to pure knowledge transfer [20–23].

The present study examined medical students’ evaluation of the new content. Potential changes in students’ knowledge of oral diseases and their associations with systemic inflammatory conditions, as well as their attitudes and perceptions toward interprofessional collaboration with dentists were assessed. As the evidence about medical students’ interests and attitudes regarding dentistry content in medical school is sparse, our results fill an important research gap. The main research questions of this study were the following:

1. Does the intervention improve students’ self-assessed competencies and increase their knowledge of oral diseases?
2. Does the new learning content positively influence medical students’ perceptions of the relevance of dental issues to the medical care of their future patients, and does it increase their recognition of the importance of interprofessional collaboration with dentists?
3. How do medical students receive the new learning content that addresses the interdisciplinary overlap between medicine and dentistry?
4. Is PBL implemented in the 5th year of medical study a suitable format for reinforcing prior knowledge and facilitating the acquisition of new content related to oral health?

## Methods

### Curricular intervention

Medical education in Germany lasts 6 years. It is divided into preclinical years (years 1 and 2), clinical years (years 3 to 5), and the final clinical year (year 6), with each section culminating in a major exam. Most German medical schools are public. More information on undergraduate medical education in Germany is provided by Chenot [24].

At the Leipzig University, all medical students in years 3 to 5 (clinical years) participate in an annual PBL course covering the topics of infectiology/immunology (year 3), emergency medical care (year 4), and geriatrics (year 5). In the 5th year, this course is called “Medicine of Aging and the Aging Person”. The participating students work independently in small tutor groups with up to nine students and one moderating tutor to acquire knowledge using clinical case stories. In the summer semester of 2021, the tutor groups were conducted in live online sessions due to the COVID-19-related restrictions in higher education in Germany [25]. Each clinical case is worked on over the course of two days, with a 90-minute live session plus individual preparation and follow-up.

All tutors are postgraduate staff from the medical faculty, with most of them being doctors or biomedical researchers. Only academic staff who successfully completed a one-week course in medical education can serve as tutors. This course, offered by the faculty, intensively addresses the PBL course format and prepares for the role as tutor through theory and practical exercises. In addition, mandatory information sessions will be held before the start of the course for all tutors to discuss patient cases and clarify any uncertainties.

In 2021, one of the six clinical cases was used to implement the new teaching content, which addressed the interdependence between oral health and chronic diseases, as well as the necessity for collaboration between GPs and dentists. Lectures and practical exercises on the topic complemented the course.

A detailed guide for teaching the course content was created to be used during the process. In addition, tutors and students were given a catalog of learning objectives to work through during the case workflow.

### The patient case

The new clinical case story called “A Case for Erna” was designed to introduce oral health topics to the students. The fictional main character, an older male patient who was previously healthy, was newly diagnosed with type 2 diabetes mellitus after being sent to the GP for general discomfort by his worried wife, “Erna”. A referral to the dentist revealed severe periodontitis. In the course of the story, the situation was aggravated by a heart attack, unmasking previously unknown CVD. Both type 2 diabetes and CVD were familiar topics to the students.

The pathology of periodontitis, its diagnostics and therapy, and its possible effects on systemic inflammatory diseases were newly introduced to the students. Knowledge about the examination of the oral cavity and the assessment of the dental status was also imparted. Students were trained to identify high-risk patients who would benefit from closer dental check-ups due to their underlying diseases and were encouraged to discuss the importance of dentistry in their own medical practice.

### Sampling and design

The effect of the new teaching content was evaluated using an observational study design. Fifth year medical students (*n* = 348) at the Leipzig University who participated in the mandatory PBL course in geriatrics in the summer semester of 2021 were invited to participate in the survey before and after the intervention to allow for pre-post comparisons. An email with a link to the pre-survey was sent centrally to all students two days before the new case session started. The opportunity to participate in the pre-survey ended when the work on the new teaching content began. An email containing the link to the follow-up survey was sent to the same students after the new case sessions were completed and this survey was available for completion within 24 days. At the Leipzig University, self-generated personal ID codes were used to anonymously assign the corresponding pre and post questionnaires. Only students who completed both questionnaires were included in the final analysis.

In addition to the pre-post survey at the Leipzig University, a comparison group of *n* = 217 students in the same year at another university (Martin Luther University Halle-Wittenberg) without corresponding course content was invited to participate in the survey once. Immediately before a mandatory exam at the end of the summer semester of 2021, students from the comparison group received a paper-based questionnaire.

Students in Halle were taught according to their universities’ standard curriculum, which does not include any PBL courses. The course “geriatrics” is taught in the form of lectures, seminars, and clinical visits. Apart from the specific design of the course formats, which universities can shape individually, studies at both universities are subject to the German licensing regulations for doctors [26]. These do not require any specific dental training.

At both universities, student participation in the evaluation was voluntary. The anonymously collected data did not allow any conclusions to be drawn about individuals. The data used in the code did not allow researchers to draw any conclusions about students’ personal data, so anonymity was maintained even when matching the data sets. Only the research team had access to the data.

### Questionnaires

The questionnaires, as well as the case story, were self-developed by a multi-professional team experienced in educational research, consisting of GPs, dentists, a medical student, and a psychologist. Some of the items used were adapted from previous studies for the purpose of later comparability [27, 28]. To ensure comprehensibility and face validity, pre-tests were conducted in the form of think-aloud protocols with five medical students who did not participate in the PBL course at the Leipzig University. This process led to minor adjustments. An English translation of the questionnaires and expected answers for the knowledge tests can be found in Supplement 1.

#### Pre-questionnaire at the Leipzig University

The pre-questionnaire at the Leipzig University contained several parts. First, relevant socio-demographic information was requested. Second, students were asked to rate their level of knowledge and confidence in dealing with oral diseases based on pre-formulated statements using a scale from 1 (does not apply) to 10 (fully applies). To objectify this self-assessment, participants were presented with a compilation of clinical pictures to rate on two criteria: (1) the risk of an increased progression of dental diseases in the presence of selected medical diseases and (2) the risk of developing complications related to the selected medical diseases after dental interventions. Students assessed the risk on a scale from 1 to 10 (1 = lowest risk, 10 = highest risk). Medical conditions, both with and without a known association with periodontitis, were selected according to a previous study by Schmalz et al. [28]. This task was supplemented by 10 multiple-choice questions on periodontitis. Each question had four possible answer options, with only one being correct. A maximum of 10 points (one for each correct answer) could be reached.

Third, the students’ perceptions and attitudes towards medical-dental collaboration and its importance were assessed using pre-formulated statements that could be rated on a scale from 1 (does not apply) to 10 (fully applies).

#### Post-questionnaire at the Leipzig University

The post-questionnaire at the Leipzig University contained the same questions as the pre-questionnaire, with some modifications as follows. The socio-demographic part was omitted, as individual ID codes linked pre- and post-questionnaires to each other. Additionally, a detailed post-hoc evaluation of the new case was requested in the form of students’ agreement with respective statements using 4-point scales (totally agree, rather agree, rather disagree, totally disagree). Finally, participants were asked about their main insights gained from the new case and were given the opportunity to provide feedback and suggestions for improvement regarding the new case work in a free-text format.

#### Questionnaire at the Martin Luther University Halle-Wittenberg (comparison group)

The questionnaire used for the survey at the Martin Luther University Halle-Wittenberg (comparison group) was the same as the pre-questionnaire used at the Leipzig University.

### Data analysis

#### Statistical analysis

Data were analyzed using IBM^®^ SPSS^®^ Statistics 29 (IBM Corp.). Continuous variables were presented as mean ± SD, and frequencies were presented as %_valid_ (n_absolute_/n_valid_), considering missing values for single items. In addition to a descriptive analysis of the data, chi-square (χ2) tests were used to compare frequency distributions between independent groups. Due to the absence of a normal distribution of the respective variables within the different groups according to Kolmogorov-Smirnov tests, Mann-Whitney U tests were used to compare differences in central tendency between independent groups, and Wilcoxon signed-rank tests were used for dependent samples (pre-post analysis). Statistical significance was assumed at *p* < 0.05.

#### Qualitative analysis of students’ main insights from the PBL case

The collected qualitative data (free text answers) were analyzed based on Mayring’s principle of qualitative content analysis [29].

First, two raters (a member of the research team and an analytical IT specialist) developed categories independently by considering all the given answers and using an inductive analytical approach. After comparing the categories, the raters agreed on the differences and together assigned the students’ answers to the consented category system. In the last step, another independent rater was asked to assign the answers once again to the consented category system. The inter-rater agreement between the consented assignment and the independent rater was 85.0%, which can be considered a good result indicating sufficient objectivity in the assignment.

## Results

At the Leipzig University, 164 pre-questionnaires and 145 post-questionnaires were collected. This resulted in a response rate of 35.9%, with 125 of 348 students completing both pre- and post-questionnaires. At the Martin Luther University Halle-Wittenberg (comparison group), 86.6% of students responded, and 188 analyzable questionnaires were returned out of 217 invited participants. Sociodemographic data showed no significant structural differences between the student groups surveyed at both universities, indicating comparability.

### Sample description

Percentages, means, and standard deviations for sample characteristics can be found in Table 1.

**Table 1:** Sociodemographic data - comparison between intervention group (Leipzig) and comparison group (Halle)

	Intervention group	Comparison group	*p*	Total
N (%)	125/348 (35.9%)	188/217 (86.6%)		313
Age mean ± SD	25.7 ± 3.1	25.5 ± 3.0	0.750	
Female n/n_valid_ (%)	85/124 (68.5%)	138/187 (73.8%)	0.424	
Semester* mean ± SD	10.0 ± 0.4	10.0 ± 0.3	0.140	
Dentist or GPs among family members				
N	125	183		308
Dentist n (%)	11 (8.8%)	11 (6.0%)		22 (7.1%)
GP n (%)	7 (5.6%)	16 (8.7%)		23 (7.5%)
Both n (%)	5 (4.0%)	5 (2.7%)		10 (3.2%)
None n (%)	102 (81.6%)	151 (82.5%)		253 (82.1%)
			0.539	
Dentist or GPs among friends or acquaintances				
N	125	187		312
Dentist n (%)	13 (10.4%)	12 (6.4%)		25 (8.0%)
GP n (%)	17 (13.6%)	32 (17.1%)		49 (15.7%)
Both n (%)	18 (14.4%)	31 (16.6%)		49 (15.7%)
None n (%)	77 (61.6%)	112 (59.9%)		189 (60.6%)
			0.506	
Mainly grew up in				
N	124	184		308
Major city n (%)	40 (32.3%)	76 (41.3%)		116 (37.7%)
Small city n (%)	41 (33.1%)	57 (31.0%)		98 (31.8%)
Rural area n (%)	43 (34.7%)	51 (27.7%)		94 (30.5%)
			0.236	
Intended specialization				
N	125	188		313
General Practice n (%)	19 (15.2%)	26 (13.8%)		45 (14.4%)
Maxillofacial Surgery n (%)	0 (0.0%)	2 (1.1%)		2 (0.6%)
Other n (%)	79 (63.2%)	123 (65.4%)		202 (64.5%)
Undecided n (%)	27 (21.6%)	37 (19.7%)		64 (20.4%)
			0.782	
For me, general practice is:				
N	124	188		312
The favorite career option n (%)	17 (13.7%)	25 (13.3%)		42 (13.5%)
An imaginable career option n (%)	76 (61.3%)	97 (51.6%)		173 (55.4%)
No career option n (%)	31 (25.0%)	66 (35.1%)		97 (31.1%)
			0.155	
For me, working in the outpatient care sector is:				
N	125	187		312
The favorite career option n (%)	53 (42.4%)	77 (41.2%)		130 (41.7%)
An imaginable career option n (%)	71 (56.8%)	101 (54.0%)		172 (55.1%)
No career option n (%)	1 (0.8%)	9 (4.8%)		10 (3.2%)
			0.143	
For me, working in one’s own medical practice is:				
N	125	188		313
The favorite career option n (%)	40 (32.0%)	51 (27.1%)		91 (29.1%)
An imaginable career option n (%)	74 (59.2%)	124 (66.0%)		198 (63.3%)
No career option n (%)	11 (8.8%)	13 (6.9%)		24 (7.7%)
			0.472	
Vocational training started and/or finished prior to medical studies				
N	124	188		312
Medical/nursing without dental reference n (%)	35 (28.2%)	39 (20.7%)		74 (23.7%)
Medical/nursing with dental reference n (%)	1 (0.8%)	1 (0.5%)		2 (0.6%)
Another n (%)	1 (0.8%)	3 (1.6%)		4 (1.3%)
None	87 (70.2%)	145 (77.1%)		232 (88.2%)
			0.155	
Other studies started and/or finished prior to medical studies				
N	125	188		313
With medical/scientific reference n (%)	6 (4.8%)	15 (8.0%)		21 (6.7%)
If yes, dentistry? n/n_valid_ (%)	0/6 (0.0%)	1/14 (7.1%)		1/20 (0.1%)
Without medical/scientific reference n (%)	10 (8.0%)	6 (3.2%)		16 (5.1%)
None	109 (87.2%)	167 (88.8%)		276 (88.2%)
			0.103	

_*Semester number out of 12 total semesters in the program_

### Self-assessment of individual skills and knowledge on oral health

**Fig. 1:**
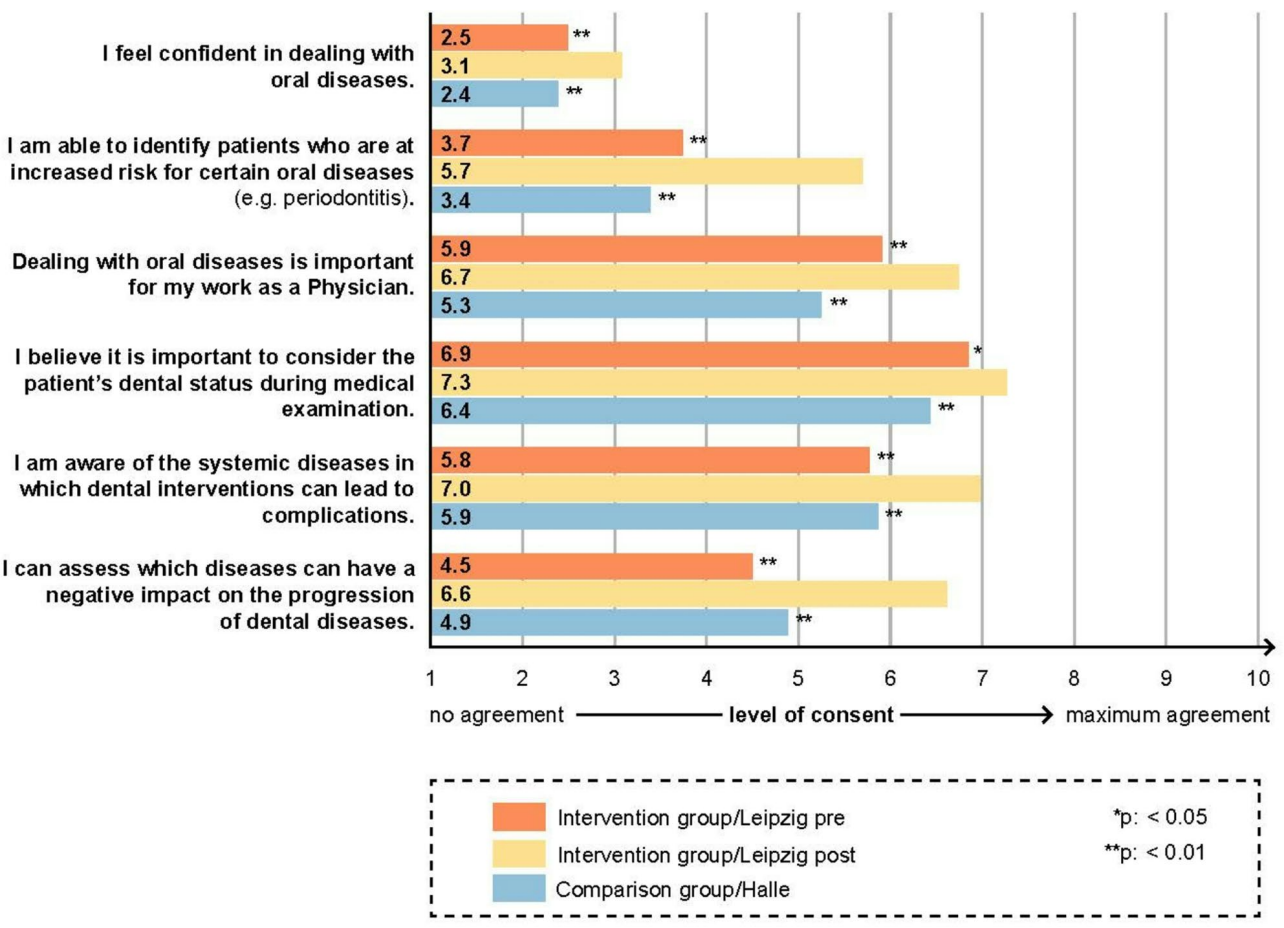
Medical students’ self-assessed competencies regarding oral diseases: Pre-intervention vs. post-intervention and post-intervention vs. comparison group. Statistically significant differences pre- vs. post-intervention as well as post-intervention vs. comparison group are marked with * for *p* < 0.05 and ** for *p* < 0.01 at the end of the corresponding bars

Figure 1 shows pre-post data and comparisons for students’ self-assessments regarding their abilities in dealing with oral diseases. Students from the intervention group scored significantly higher in items on confidence in dealing with dental pathologies and on increased awareness of medical-dental interrelationships post vs. pre intervention and compared to the comparison group.

### Students’ assessment of the bi-directional links between oral diseases and medical conditions

**Fig. 2:**
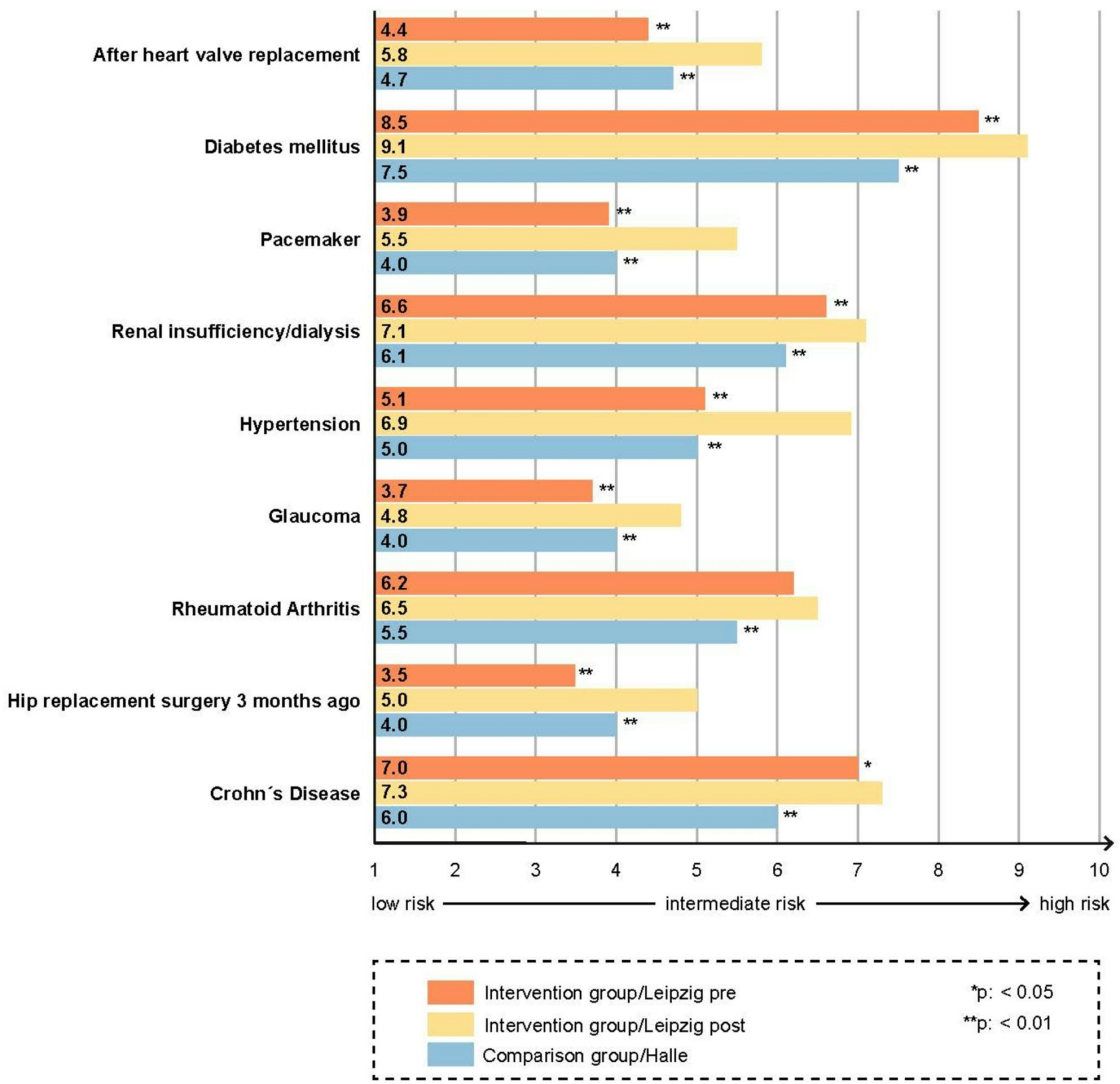
Students’ assessment of oral disease progression risk under selected medical conditions, pre-intervention vs. post-intervention and post-intervention vs. comparison group. Statistically significant differences pre- vs. post-intervention as well as post-intervention vs. comparison group are marked with * for *p* < 0.05 and ** for *p* < 0.01 at the end of the corresponding bars

**Fig. 3:**
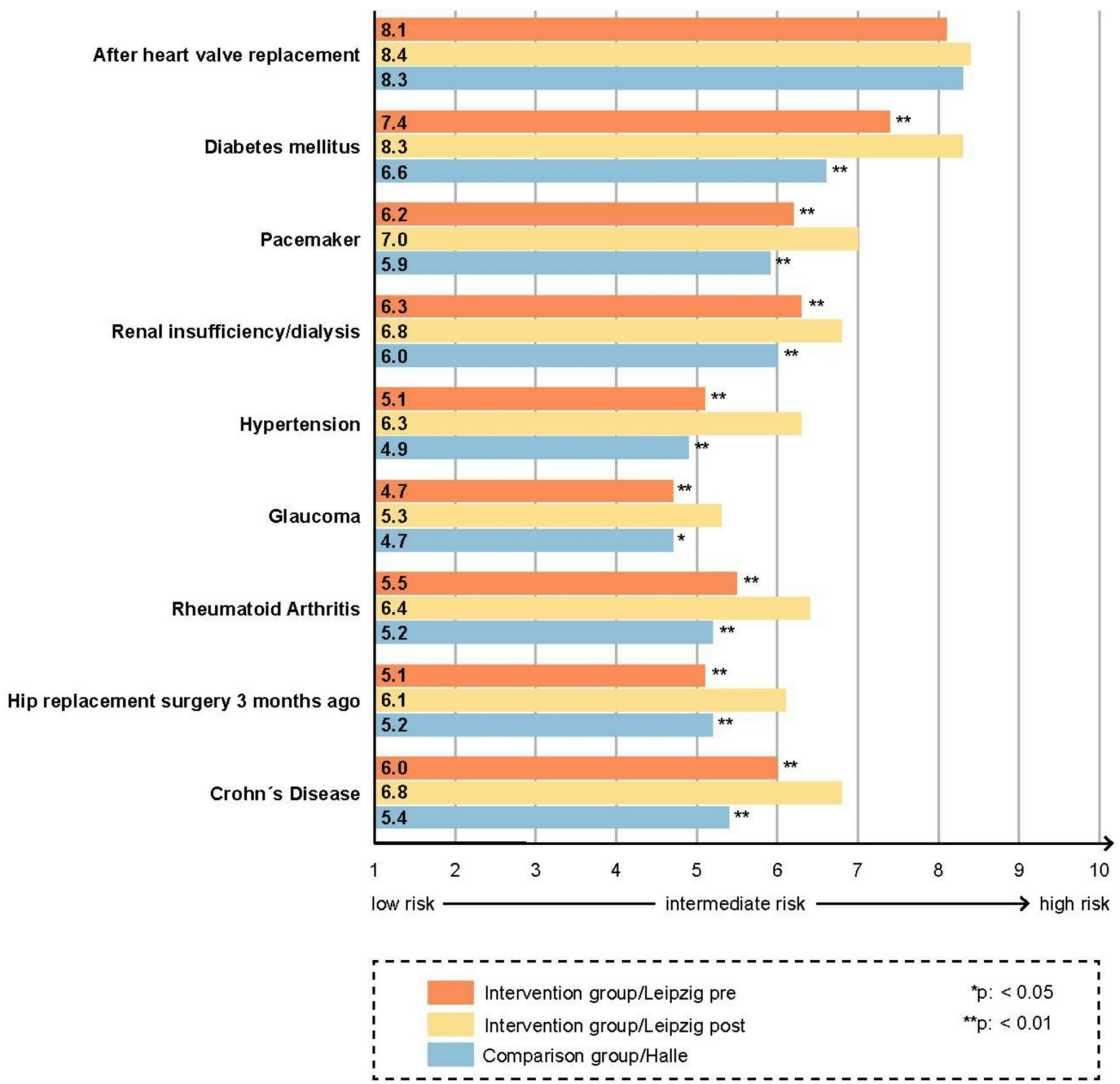
Students’ assessment of complication risk in selected medical conditions after dental interventions, pre-intervention vs. post-intervention and post-intervention vs. comparison group. Statistically significant differences pre- vs. post-intervention as well as post-intervention vs. comparison group are marked with * for *p* < 0.05 and ** for *p* < 0.01 at the end of the corresponding bars

Students in the intervention group showed a significant increase in agreement with the statement that the risk of progression of oral diseases was higher in the presence of selected medical conditions (Fig. 2). They also expressed stronger agreement that the risk of complications related to the medical diseases increases after dental interventions (Fig. 3). This applied to both correct and incorrect associations.

### Results of the multiple-choice test

A maximum of 10 points (1 point for each correctly answered question) could be reached. Students achieved significantly higher scores after the intervention. This could be seen in pre – post comparison (pre: 4.1 ± 1.4 vs. post: 4.9 ± 1.5; *p* < 0.001) as well as in contrast to the comparison group (comparison: 3.9 ± 1.5 vs. post: 4.9 ± 1.5; *p* < 0.001).

### Students’ perception of general aspects regarding the field of dentistry

After the curricular intervention, Leipzig students agreed more than they had before, and more than students from Halle-Wittenberg (comparison group), with the following statements:

“I think collaboration between dentists and GPs is important” (Leipzig post: 8.3 ± 1.5; vs. Leipzig pre: 7.4 ± 1.8, *p* < 0.001; vs. comparison: 6.7 ± 2.4, *p* < 0.001). “Depending on individual risk factors, the dental status should also be recorded as part of the intraoral examination by the GP” (Leipzig post: 7.9 ± 1.9; vs. pre: 7.1 ± 2.2, *p* = 0.001; vs. comparison: 5.8 ± 2.5, *p* < 0.001). “Oral health and general health are closely connected” (Leipzig post: 8.3 ± 1.6; vs. pre: 8.0 ± 1.7, *p* = 0.005; vs. comparison: 7.0 ± 2.3, *p* < 0.001).

“I would like to maintain closer contact with my dental colleagues in my future professional life” (Leipzig post: 6.4 ± 2.3; vs. pre: 5.4 ± 2.3, *p* < 0.001; vs. comparison: 5.1 ± 2.2, *p* < 0.001).

The results also indicated a perceived separation between dentistry and medicine during the preclinical study phase. Leipzig students rated the statement “I already perceived a clear separation between dental and medicine students in the preclinical phase” rather high (7.9 ± 2.3). Contact with oral health topics in medical studies was reported as low (“I was taught about dental clinical pictures in my previous studies”, 3.5 ± 2.1).

### Post-hoc evaluation of the new teaching content

The post-hoc evaluation showed that the new learning content was well received by the students. The overwhelming majority of the responders agreed (chose the terms “totally agree” or “rather agree”) that they gained new insights through the case (111/124 = 89.5%) and that their personal perception of the importance of interprofessional collaboration with dentists was positively influenced (109/124 = 87.9%). Two out of five students (50/125 = 40.0%) agreed that their interest in dentistry was increased. More than half of the participants (72/125 = 57.6%) felt that the time in the course was well chosen to place the new content. The PBL course on geriatrics was seen as a suitable format by the majority of students (107/124 = 86.3%). Overall, 66.4% (83/125) enjoyed working on the case, and 85.5% (106/124) stated that the case was valuable for their studies.

### Qualitative analysis of students’ free text answers

The main insights that emerged from the case processing *(“Which main insights do you take away from the case?*”) are displayed in Table 2. A total of 61 Students (48.8% of the sample) provided 94 statements. The maximum number of statements provided by an individual student was 3, and no students provided more than one statement per category.

**Table 2:** “Which main insights do you take away from the case?”

Category title	Number of statements *n* (%)	“Which main insights do you take away from the case?” Examples of student responses
Interrelationships/ overlaps	41 (43.6%)	“The awareness that dental diseases are directly related to systemic diseases and that I as a doctor should therefore also consider dental health to ensure holistic care.”
Importance of interdisciplinary collaboration	15 (16.0%)	“Cooperation between general practitioners and dentists is essential and important.”
Assessment of the dental status	13 (13.8%)	“That you should always check the dental status of a patient and not completely disregard it.”
Relevance of dental knowledge	11 (11.7%)	“As a human medicine specialist, you should know the basics of dentistry.”
Lack of teaching dental contents in medical studies	6 (6.4%)	“When we study human medicine, we learn almost nothing about dental diseases.”
The perception of own deficits with regard to dental knowledge	6 (6.4%)	“The fact that I know nothing about dentistry”
Importance of targeted questions during the medical history taking	2 (2.1%)	“You really have to pepper patients with questions so that they tell you everything (keyword: do they have chest pain? ).”

When asked about the positive aspects of the new teaching content (question: “*What did you like in particular?”*), 74 students provided 79 statements. Students provided no more than 4 statements. No more than 2 statements per student were assigned to the same category.

Of them, 24 statements (30.4%) referred to the positive perception of integrating dentistry into undergraduate medical studies.

*Example:“That dentistry has found its way into a PBL case.”*

Another 18 (22.8%) statements mentioned specific content points that had made a positive impression on them.

*Example: “The combination of the familiar topic of diabetes with the new topic of periodontitis and the mutual interactions (…).”*

In addition, 14 (17.7%) statements highlighted the relevance for medical practice.

*Example: “Medical students have no idea about this, although dental health can have such an impact on systemic diseases (…).”*

Altogether, 108 statements from 67 respondents (max. 4 statements per student, max. 2 statements per category) addressed suggestions for improvement (*question: “What do you think should be improved?”*).

Additional teaching formats/lectures were considered missing in 23 (21.3%) statements.

*Example: “These topics should also be covered in the PBL3 lectures. So then also a teaching content on periodontitis (…).”*

A lack of further illustrative material was mentioned in 17 (15.7%) statements.

*Example: “New theoretical content should be provided as an overview for reference.” *

The lack of prior knowledge was named as an obstacle to the discussion in 14 (13.0%) statements.

*Example: “The lack of basic knowledge often led to collective helplessness (…)”*

A complete overview of positive and negative aspects of the new teaching content can be found in Supplement 2.

## Discussion

### Summary of the main findings

The study provides indications that the new learning content on oral health enhances students’ self-assessed competencies and increases their knowledge. Group differences were found in both the pre-post comparison and when compared to a group that did not attend the sessions.

Further, the new teaching content seems to positively influence medical students’ perceptions of the importance of dental issues and the need for collaboration with dental colleagues. Overall, the students welcomed the integration of oral health topics. The vast majority agreed that the course was valuable and that they had gained new insights through the case study. PBL was seen as a suitable format by most of the students. Further studies are required to confirm these results.

#### Literature Comparison

Several studies located in the U.S. described teaching interventions addressing oral health knowledge and skills for undergraduate medical students [30–32]. All of them referred to the Smiles for Life Curriculum [33], a national resource that provides educational material for the promotion of oral health in primary care. Silk et al. conducted half-day workshops for third-year students, including lectures and hands-on sessions [30]. Morel et al. supplemented the teaching sessions with a three-day clinical clerkship [31]. Park et al. surveyed an intervention for second-year students in a patient-doctor-course, including lectures, flipped-classroom materials, and a PBL session with a hands-on component. Medicine and dentistry students were taught together [32].

Studies that included a pre-post-knowledge test reported an increase in knowledge [30, 31], although the effect declined in a re-test six months later [30]. U.S. students scored much higher than the students in our study in both pre- and post-knowledge tests. Despite the fact that medical studies in Germany and the U.S. show important structural differences, which hinders comparability, this is reflected by our students’ comments in the free text part, where they complained about the lack of preparation and knowledge necessary for the case discussions.

With regard to the self-assessment, students reported feeling more comfortable conducting oral examinations, identifying patients at risk for oral diseases, and providing patients with basic information on oral health topics [31, 32], which aligns with our results. Interestingly, all studies, including ours, found that medical students placed great importance on oral health and collaboration with dentists, even before the intervention. This could be due to a genuine interest in the topic or could be influenced by selective participation in the study, social desirability, or expectancy. This went so far that students in our study consistently attested high mutual influence of oral health issues and selected medical conditions. This would have been particularly accurate in relation to chronic inflammatory systemic diseases, but not in cases of others (e.g., glaucoma). Overall, we succeeded in raising awareness of the dental-medical linkages, although we acknowledge the need for a more in-depth theoretical underpinning of our course content.

The poor results of our students’ knowledge raise concerns. Although the questions were aligned with the teaching content and learning objectives, the two-day PBL patient case might have been too short to address all topics with due intensity. We believe that the results should be taken as a call to integrate more oral health topics into the curriculum in the future, possibly in the form of a learning spiral that enables repeated take up and deepening of oral health topics from preclinical to clinical years. This might also tackle the poor sustainability demonstrated by Silk et al. [30], which could not be confirmed in our study due to differences in the study design. It is consistent, though, with results from a previous study with dental students, that succeeded in raising awareness of the importance of identifying patients at risk for complications, but achieved only mixed results in a knowledge test three months after the course [27]. Knowledge test results improved, however, in a second study that introduced a structured tool and taught its application [28]. The author team, some of whom participated in the design of this study, concluded that the use of specific, structured content in the form of the new tool helped students to better deal with the knowledge queries.

As a consequence of our study, we introduced an additional lecture and further training for tutors as a first step, both in collaboration with dental lecturers. Of course, it would also be desirable to integrate collaboration with dentistry colleagues into undergraduate medical education, for example in the form of joint courses with dental students.

All of these ideas are supported by recent U.S. expert recommendations that (among others) propose to “maximize touchpoints” throughout undergraduate education, include skills training, and focus on oral-systemic connections and bi-directional learning with dentists as important components for successful integration of oral health into medical studies [32]. Interdisciplinary approaches, in particular, lead to an increase in knowledge and attitudes of students and should also be considered in future curriculum development [34, 35].

However, despite all the enthusiasm for new learning content, it must be remembered that it is not only oral health topics that are entering medical studies. In an increasingly complex medical world, oral health topics compete with a broad range of other demands on medical students. Whether and which additional content and formats are offered to students is therefore a result of careful consideration of the costs and benefits and may also lead to cutbacks in the existing curriculum.

#### Strengths and limitations

This study investigated an innovative topic that has received little attention in many countries worldwide, although there have been numerous calls for action [16, 17]. The sample size was considerable, and the inclusion of a comparison group from a second medical school enhanced its explanatory power. Thus, the study design allowed pre-post analysis and comparison with students who did not receive training on oral health. Furthermore, the survey collected both qualitative and quantitative data. The quantitative data included not only students’ self-assessment of acquired competencies, but also objective knowledge tests.

This study has limitations, too. Due to the exploratory nature and the specific context of evaluating teaching interventions with students in undergraduate medical education, no sample size calculation was performed. Furthermore, the questionnaire was self-developed and based on non-validated items. This should be kept in mind when interpreting our results.

The response rate in the intervention group was low. This could have resulted in selection bias by including students who were particularly interested in the topic of oral health and may have especially benefited from the intervention. Furthermore, students in the comparison group came from another university in a different federal state. While our data showed no significant differences in socio-demographic variables, comparability may have been affected by differences in the curriculum and in the timing and circumstances of the survey. However, uniform regulations on medical studies in Germany (“Approbationsordnung für Ärzte” – Licensing regulations for physicians [26]) make substantial curricular differences unlikely. Due to the study design, we could only measure the short-term effects of a one-off intervention. The extent to which these effects persist in the long term and their influence on the participants’ future medical practices remains to be investigated.

Finally, the quality of PBL courses also depends on the composition of the student group and the tutor’s interest and didactic skills. Consequently, processing the new teaching content in the course groups may have differed, which had a corresponding effect on the students’ assessments and learning gains. However, this reflects “real life” conditions in undergraduate (medical) education and should have been limited by the tutors’ didactic training and preparation sessions.

## Conclusion

The results of this pilot study suggest that the new teaching format, a PBL case story addressing dental-medical overlap and collaboration, is suitable to contribute to imparting knowledge, generating interest in the topic, and raising awareness of dental-medical interlinkages. PBL was seen as a suitable format by the participants. Although medical students were generally open to the topic of dentistry, the study indicates significant gaps in their knowledge and skills. Whether these gaps can be filled by additional lectures remains to be seen, but longitudinal anchorage and interdisciplinary approaches should be the focus of further research. At the same time, data on long-term outcomes and the practical application of knowledge gained from the course in future professional life is urgently required and could be the starting point for further studies.

## Supplementary Information

Supplementary Material 1.

Supplementary Material 2.

## Data Availability

The datasets are available from the corresponding author on reasonable request.

## Abbreviations

CVD: Cardiovascular disease
GP: General Practitioner
Max.: Maximum
PBL: Problem-based learning
U.S.: United States of America
Vs.: Versus
