# Supplementary material for: Raising medical students’ awareness for the interdependence between oral health and systemic diseases - evaluation of a problem-based learning intervention: an exploratory pilot study
Source: BMC Prim Care. 2026 Apr 24;27:161. doi: 10.1186/s12875-026-03323-4 (PMC13109874; doi:10.1186/s12875-026-03323-4)
Supplement: Supplementary file 1 — Supplementary Material 1. [file 12875_2026_3323_MOESM1_ESM.docx]

**Supplement 1**

1. **Questionnaire Leipzig University (pre) and Martin Luther University Halle-Wittenberg (comparison group)**

| 1. **Sociodemographic information** | | |
| --- | --- | --- |
| Age: _____years | Gender: 〇 Male 〇 Female 〇 Divers | Semester: ______ |
| Dentist or GPs among family members: | 〇 Dentist 〇 GP 〇 both | 〇 none |
| Dentist or GPs among friends or acquaintances | 〇 Dentist 〇 GP. 〇 both | 〇 none |
| I mainly grew up in | 〇 Major city 〇 small city 〇 rural area | |

| **Intended specialization:** | 〇 General Practice  〇 Maxillofacial Surgery | 〇 other: | 〇 undecided |
| --- | --- | --- | --- |
| **For me, general practice is:** | 〇 The favorite career option | 〇 An imaginable career option | 〇 No career option |
| **For me, working in the outpatient care sector is:** | 〇 The favorite career option | 〇 An imaginable career option | 〇 No career option |
| **For me, working in one’s own medical practice is:** | 〇 The favorite career option | 〇 An imaginable career option | 〇 No career option |
| **I have started and/or finished**  **vocational training prior to medical studies** | 〇 yes:  〇 medical/nursing without dental reference  〇 medical/nursing with dental reference | 〇 yes, not medical/nursing | 〇 None |
| **I have started and/or finished studies prior to medical studies** | 〇 Yes, with medical/scientific reference  If yes, dentistry? 〇 yes 〇 no | 〇 Yes, without medical/scientific reference | 〇 none |

| 1. **General assessment (1 - does not apply, 10 - fully applies)** | | | | | | | | | | |
| --- | --- | --- | --- | --- | --- | --- | --- | --- | --- | --- |
|  | 1 | 2 | 3 | 4 | 5 | 6 | 7 | 8 | 9 | 10 |
| I think collaboration between dentists and GPs is important. | 〇 | 〇 | 〇 | 〇 | 〇 | 〇 | 〇 | 〇 | 〇 | 〇 |
| Students of medicine and dentistry should attend more courses together during their studies. | 〇 | 〇 | 〇 | 〇 | 〇 | 〇 | 〇 | 〇 | 〇 | 〇 |
| For the optimal course of treatment of many chronic diseases, it is important that the family doctor is informed about the dental history of his patient. | 〇 | 〇 | 〇 | 〇 | 〇 | 〇 | 〇 | 〇 | 〇 | 〇 |
| Depending on individual risk factors, the dental status should also be recorded as part of the intraoral examination by the GP. | 〇 | 〇 | 〇 | 〇 | 〇 | 〇 | 〇 | 〇 | 〇 | 〇 |
| Dentists can identify patients at risk of developing various chronic diseases. | 〇 | 〇 | 〇 | 〇 | 〇 | 〇 | 〇 | 〇 | 〇 | 〇 |
| The treatment of dental pathologies is important for the sustainable treatment of various chronic diseases.s | 〇 | 〇 | 〇 | 〇 | 〇 | 〇 | 〇 | 〇 | 〇 | 〇 |
| Dentistry is an interesting topic for me. | 〇 | 〇 | 〇 | 〇 | 〇 | 〇 | 〇 | 〇 | 〇 | 〇 |
| Oral health and general health are closely connected**.** | 〇 | 〇 | 〇 | 〇 | 〇 | 〇 | 〇 | 〇 | 〇 | 〇 |
| I would like to maintain closer contact with my dental colleagues in my future professional life. | 〇 | 〇 | 〇 | 〇 | 〇 | 〇 | 〇 | 〇 | 〇 | 〇 |
| I was taught about dental clinical pictures in my previous studies. | 〇 | 〇 | 〇 | 〇 | 〇 | 〇 | 〇 | 〇 | 〇 | 〇 |
| I already perceived a clear separation between dental and medicine students in the preclinical phase. | 〇 | 〇 | 〇 | 〇 | 〇 | 〇 | 〇 | 〇 | 〇 | 〇 |
| Students of human medicine should learn more about dental conditions during their studies. | 〇 | 〇 | 〇 | 〇 | 〇 | 〇 | 〇 | 〇 | 〇 | 〇 |
| I think that dental students acquire a comprehensive knowledge of systemic diseases during their studies. | 〇 | 〇 | 〇 | 〇 | 〇 | 〇 | 〇 | 〇 | 〇 | 〇 |

| 1. **Self-assessment (1 - does not apply, 10 - fully applies)** | | | | | | | | | | |
| --- | --- | --- | --- | --- | --- | --- | --- | --- | --- | --- |
|  | 1 | 2 | 3 | 4 | 5 | 6 | 7 | 8 | 9 | 10 |
| I feel confident in dealing with oral diseases. | 〇 | 〇 | 〇 | 〇 | 〇 | 〇 | 〇 | 〇 | 〇 | 〇 |
| I am able to identify patients who are at an inceased risk for certain oral diseases. | 〇 | 〇 | 〇 | 〇 | 〇 | 〇 | 〇 | 〇 | 〇 | 〇 |
| Dealing with oral diseases is important for my work as a physician. | 〇 | 〇 | 〇 | 〇 | 〇 | 〇 | 〇 | 〇 | 〇 | 〇 |
| I believe it is important to consider the patient’s dental status during medical examination. | 〇 | 〇 | 〇 | 〇 | 〇 | 〇 | 〇 | 〇 | 〇 | 〇 |
| I am aware of the systemic diseases in which dental interventions can lead to complications. | 〇 | 〇 | 〇 | 〇 | 〇 | 〇 | 〇 | 〇 | 〇 | 〇 |
| I can assess which diseases can have a negative impact on the progression of dental diseases. | 〇 | 〇 | 〇 | 〇 | 〇 | 〇 | 〇 | 〇 | 〇 | 〇 |

| 1. **Assessment of selected clinical pictures (1 - lowest risk, 10 - highest risk)** | | | | | | | | | | |
| --- | --- | --- | --- | --- | --- | --- | --- | --- | --- | --- |
| For the following diseases, I estimate the risk of developing a complication (related to the underlying disease) after a dental intervention as follows: | 1 | 2 | 3 | 4 | 5 | 6 | 7 | 8 | 9 | 10 |
| After Heart valve replacement | 〇 | 〇 | 〇 | 〇 | 〇 | 〇 | 〇 | 〇 | 〇 | 〇 |
| Diabetes mellitus | 〇 | 〇 | 〇 | 〇 | 〇 | 〇 | 〇 | 〇 | 〇 | 〇 |
| Pacemaker | 〇 | 〇 | 〇 | 〇 | 〇 | 〇 | 〇 | 〇 | 〇 | 〇 |
| Renal insufficiency/dialysis | 〇 | 〇 | 〇 | 〇 | 〇 | 〇 | 〇 | 〇 | 〇 | 〇 |
| Hypertension | 〇 | 〇 | 〇 | 〇 | 〇 | 〇 | 〇 | 〇 | 〇 | 〇 |
| Glaukoma | 〇 | 〇 | 〇 | 〇 | 〇 | 〇 | 〇 | 〇 | 〇 | 〇 |
| Rheumatoid arthritis | 〇 | 〇 | 〇 | 〇 | 〇 | 〇 | 〇 | 〇 | 〇 | 〇 |
| Hip replacement surgery 3 months ago | 〇 | 〇 | 〇 | 〇 | 〇 | 〇 | 〇 | 〇 | 〇 | 〇 |
| Crohn’s disease | 〇 | 〇 | 〇 | 〇 | 〇 | 〇 | 〇 | 〇 | 〇 | 〇 |

| I estimate the risk of an increased progression of an oral disease in presence of the selected medical conditions as follows: | 1 | 2 | 3 | 4 | 5 | 6 | 7 | 8 | 9 | 10 |
| --- | --- | --- | --- | --- | --- | --- | --- | --- | --- | --- |
| After Heart valve replacement | 〇 | 〇 | 〇 | 〇 | 〇 | 〇 | 〇 | 〇 | 〇 | 〇 |
| Diabetes mellitus | 〇 | 〇 | 〇 | 〇 | 〇 | 〇 | 〇 | 〇 | 〇 | 〇 |
| Pacemaker | 〇 | 〇 | 〇 | 〇 | 〇 | 〇 | 〇 | 〇 | 〇 | 〇 |
| Renal insufficiency/dialysis | 〇 | 〇 | 〇 | 〇 | 〇 | 〇 | 〇 | 〇 | 〇 | 〇 |
| Hypertension | 〇 | 〇 | 〇 | 〇 | 〇 | 〇 | 〇 | 〇 | 〇 | 〇 |
| Glaukoma | 〇 | 〇 | 〇 | 〇 | 〇 | 〇 | 〇 | 〇 | 〇 | 〇 |
| Rheumatoide arthritis | 〇 | 〇 | 〇 | 〇 | 〇 | 〇 | 〇 | 〇 | 〇 | 〇 |
| Hip replacement surgery 3 months ago | 〇 | 〇 | 〇 | 〇 | 〇 | 〇 | 〇 | 〇 | 〇 | 〇 |
| Crohn’s disease | 〇 | 〇 | 〇 | 〇 | 〇 | 〇 | 〇 | 〇 | 〇 | 〇 |

| 1. **Multiple-Choice Test** |
| --- |

The term “periodontitis” describes

A. Inflammation of the gums

B. Inflammation of the periodontium

C. Inflammation of the tooth root

D. A general inflammation of the oral mucosa

The basic periodontitis treatment at the dentist

A. Consists of subgingival instrumentation

B. Consists of A. and the additional administration of a systemic broad-spectrum antibiotic

C. Usually requires surgical intervention

D. Should only be performed on diabetics after successful blood sugar control

Wrong answer requested: The influence of DM on periodontitis is based on the following mechanisms

1. Activation of collagenases
2. AGEs and RAGE cascade
3. Increased HbA1c values
4. Side effect of insulin therapy

Wrong answer requested: Periodontitis and coronary heart disease

A. The link between periodontitis and CHD is scientifically proven

B. Oral surgery associated with periodontitis can accelerate foam cell formation in arterial vessels

C. Have almost identical risk factors

D. Components of odontopathogenic germs have already been detected in plaques on the coronary arteries

At-home mechanical biofilm management should consist of:

A. Brushing twice daily for 2 minutes, using mouth rinses and flossing

B. Brushing twice a day for 2 minutes, using interdental brushes or floss

C. Brushing twice a day for 2 minutes, using a tongue scraper and dental floss

D. Brush twice a day for 2 minutes and refrain from further measures

Subgingival instrumentation means

1. The administration of an antibiotic into a gingival pocket
2. The direct application of an antibiotic into the wound after a dental surgical procedure
3. The removal of biofilm by the dentist in the upper root area without opening the gums
4. The removal of biofilm in deeper root areas after their surgical exposure

The staging of periodontitis:

A. Is based on the severity of the periodontitis itself

B. Is based on A. and including risk factors

C. Provides information on expected disease progression

D. Is a new procedure that patients pay for themselves as a private medical service

Wrong answer sought: The influence of periodontitis on DM is directly due to the following mechanisms:

A. Acceleration of the destruction of the ß-cells

B. Bacteremia due to oral biofilm

C. Increase in systemic levels of CRP and TNFalpha, among others

D. Inadequate nutrition due to poor dental status

Risk factors for the development of periodontitis are:

A. Migraine, CHD, diabetes, smoking, childlessness

B. Poor oral hygiene, diabetes, diseases associated with immunodeficiency, menopause

C. Psoriasis, diabetes, smoking, poor oral hygiene

D. All of the named answers apply

Recording patients with an increased risk of complications after oral surgery

A. Is carried out with the involvement of the general practitioner

B. Is usually carried out for all patients at regular intervals by means of medical history questionnaires at the dentist

C. Only carried out by the dentist for patients with known pre-existing conditions

D. If a high-risk patient is identified, the dentist must inform the family doctor of the planned procedure

1. **Questionnaire Leipzig University (post)**

| 1. **General assessment (1 - does not apply, 10 - fully applies)** | | | | | | | | | | |
| --- | --- | --- | --- | --- | --- | --- | --- | --- | --- | --- |
|  | 1 | 2 | 3 | 4 | 5 | 6 | 7 | 8 | 9 | 10 |
| I think collaboration between dentists and GPs is important. | 〇 | 〇 | 〇 | 〇 | 〇 | 〇 | 〇 | 〇 | 〇 | 〇 |
| Students of human and dentistry should attend more courses together during their studies. | 〇 | 〇 | 〇 | 〇 | 〇 | 〇 | 〇 | 〇 | 〇 | 〇 |
| For the optimal course of treatment of many chronic diseases, it is important that the family doctor is informed about the dental history of his patient. | 〇 | 〇 | 〇 | 〇 | 〇 | 〇 | 〇 | 〇 | 〇 | 〇 |
| Depending on individual risk factors, the dental status should also be recorded as part of the intraoral examination by the GP | 〇 | 〇 | 〇 | 〇 | 〇 | 〇 | 〇 | 〇 | 〇 | 〇 |
| Dentists can identify patients at risk of developing various chronic diseases. | 〇 | 〇 | 〇 | 〇 | 〇 | 〇 | 〇 | 〇 | 〇 | 〇 |
| The treatment of dental pathologies is important for the sustainable treatment of various chronic diseases. | 〇 | 〇 | 〇 | 〇 | 〇 | 〇 | 〇 | 〇 | 〇 | 〇 |
| Dentistry is an interesting topic for me. | 〇 | 〇 | 〇 | 〇 | 〇 | 〇 | 〇 | 〇 | 〇 | 〇 |
| Oral health and general health are closely connected**.** | 〇 | 〇 | 〇 | 〇 | 〇 | 〇 | 〇 | 〇 | 〇 | 〇 |
| I would like to maintain closer contact with my dental colleagues in my future professional life | 〇 | 〇 | 〇 | 〇 | 〇 | 〇 | 〇 | 〇 | 〇 | 〇 |
| Students of human medicine should learn more about dental conditions during their studies. | 〇 | 〇 | 〇 | 〇 | 〇 | 〇 | 〇 | 〇 | 〇 | 〇 |
| I think that dental students acquire a comprehensive knowledge of systemic diseases during their studies. | 〇 | 〇 | 〇 | 〇 | 〇 | 〇 | 〇 | 〇 | 〇 | 〇 |

| 1. **Self-assessment (1 - does not apply, 10 - fully applies)** | | | | | | | | | | |
| --- | --- | --- | --- | --- | --- | --- | --- | --- | --- | --- |
|  | 1 | 2 | 3 | 4 | 5 | 6 | 7 | 8 | 9 | 10 |
| I feel confident in dealing with dental conditions. | 〇 | 〇 | 〇 | 〇 | 〇 | 〇 | 〇 | 〇 | 〇 | 〇 |
| I am able to identify patients who are at an inceased risk for certain dental pathologies. | 〇 | 〇 | 〇 | 〇 | 〇 | 〇 | 〇 | 〇 | 〇 | 〇 |
| Dealing with dental conditions is important for my work as a physician. | 〇 | 〇 | 〇 | 〇 | 〇 | 〇 | 〇 | 〇 | 〇 | 〇 |
| I believe it is important to consider the patient’s dental status during medical examination. | 〇 | 〇 | 〇 | 〇 | 〇 | 〇 | 〇 | 〇 | 〇 | 〇 |
| I am aware of the systemic diseases in which dental interventions can lead to complications. | 〇 | 〇 | 〇 | 〇 | 〇 | 〇 | 〇 | 〇 | 〇 | 〇 |
| I can assess which diseases can have a negative impact on the progression of dental diseases. | 〇 | 〇 | 〇 | 〇 | 〇 | 〇 | 〇 | 〇 | 〇 | 〇 |

| 1. **Assessment of selected clinical pictures (1 - lowest risk, 10 - highest risk)** | | | | | | | | | | |
| --- | --- | --- | --- | --- | --- | --- | --- | --- | --- | --- |
| For the following diseases, I estimate the risk of developing a complication (related to the underlying disease) after a dental intervention as follows: | 1 | 2 | 3 | 4 | 5 | 6 | 7 | 8 | 9 | 10 |
| After Heart valve replacement | 〇 | 〇 | 〇 | 〇 | 〇 | 〇 | 〇 | 〇 | 〇 | 〇 |
| Diabetes mellitus | 〇 | 〇 | 〇 | 〇 | 〇 | 〇 | 〇 | 〇 | 〇 | 〇 |
| Pacemaker | 〇 | 〇 | 〇 | 〇 | 〇 | 〇 | 〇 | 〇 | 〇 | 〇 |
| Renal insufficiency/dialysis | 〇 | 〇 | 〇 | 〇 | 〇 | 〇 | 〇 | 〇 | 〇 | 〇 |
| Hypertension | 〇 | 〇 | 〇 | 〇 | 〇 | 〇 | 〇 | 〇 | 〇 | 〇 |
| Glaukoma | 〇 | 〇 | 〇 | 〇 | 〇 | 〇 | 〇 | 〇 | 〇 | 〇 |
| Rheumatoid arthritis | 〇 | 〇 | 〇 | 〇 | 〇 | 〇 | 〇 | 〇 | 〇 | 〇 |
| Hip replacement surgery 3 months ago | 〇 | 〇 | 〇 | 〇 | 〇 | 〇 | 〇 | 〇 | 〇 | 〇 |
| Crohn’s disease | 〇 | 〇 | 〇 | 〇 | 〇 | 〇 | 〇 | 〇 | 〇 | 〇 |

| I estimate the risk of an increased progression of an oral disease in presence of the selected medical conditions as follows: | 1 | 2 | 3 | 4 | 5 | 6 | 7 | 8 | 9 | 10 |
| --- | --- | --- | --- | --- | --- | --- | --- | --- | --- | --- |
| After Heart valve replacement | 〇 | 〇 | 〇 | 〇 | 〇 | 〇 | 〇 | 〇 | 〇 | 〇 |
| Diabetes mellitus | 〇 | 〇 | 〇 | 〇 | 〇 | 〇 | 〇 | 〇 | 〇 | 〇 |
| Pacemaker | 〇 | 〇 | 〇 | 〇 | 〇 | 〇 | 〇 | 〇 | 〇 | 〇 |
| Renal insufficiency/dialysis | 〇 | 〇 | 〇 | 〇 | 〇 | 〇 | 〇 | 〇 | 〇 | 〇 |
| Hypertension | 〇 | 〇 | 〇 | 〇 | 〇 | 〇 | 〇 | 〇 | 〇 | 〇 |
| Glaukoma | 〇 | 〇 | 〇 | 〇 | 〇 | 〇 | 〇 | 〇 | 〇 | 〇 |
| Rheumatoide arthritis | 〇 | 〇 | 〇 | 〇 | 〇 | 〇 | 〇 | 〇 | 〇 | 〇 |
| Hip replacement surgery 3 months ago | 〇 | 〇 | 〇 | 〇 | 〇 | 〇 | 〇 | 〇 | 〇 | 〇 |
| Crohn’s disease | 〇 | 〇 | 〇 | 〇 | 〇 | 〇 | 〇 | 〇 | 〇 | 〇 |

| 1. **Multiple-Choice Test** |
| --- |

The term “periodontitis” describes

A. Inflammation of the gums

B. Inflammation of the periodontium

C. Inflammation of the tooth root

D. A general inflammation of the oral mucosa

The basic periodontitis treatment at the dentist

A. Consists of subgingival instrumentation

B. Consists of A. and the additional administration of a systemic broad-spectrum antibiotic

C. Usually requires surgical intervention

D. Should only be performed on diabetics after successful blood sugar control

Wrong answer requested: The influence of DM on periodontitis is based on the following mechanisms

1. Activation of collagenases
2. AGEs and RAGE cascade
3. Increased HbA1c values
4. Side effect of insulin therapy

Wrong answer requested: Periodontitis and coronary heart disease

A. The link between periodontitis and CHD is scientifically proven

B. Oral surgery associated with periodontitis can accelerate foam cell formation in arterial vessels

C. Have almost identical risk factors

D. Components of odontopathogenic germs have already been detected in plaques on the coronary arteries

At-home mechanical biofilm management should consist of:

A. Brushing twice daily for 2 minutes, using mouth rinses and flossing

B. Brushing twice a day for 2 minutes, using interdental brushes or floss

C. Brushing twice a day for 2 minutes, using a tongue scraper and dental floss

D. Brush twice a day for 2 minutes and refrain from further measures

Subgingival instrumentation means

1. The administration of an antibiotic into a gingival pocket
2. The direct application of an antibiotic into the wound after a dental surgical procedure
3. The removal of biofilm by the dentist in the upper root area without opening the gums
4. The removal of biofilm in deeper root areas after their surgical exposure

The staging of periodontitis:

A. Is based on the severity of the periodontitis itself

B. Is based on A. and including risk factors

C. Provides information on expected disease progression

D. Is a new procedure that patients pay for themselves as a private medical service

Wrong answer sought: The influence of periodontitis on DM is directly due to the following mechanisms:

A. Acceleration of the destruction of the ß-cells

B. Bacteremia due to oral biofilm

C. Increase in systemic levels of CRP and TNFalpha, among others

D. Inadequate nutrition due to poor dental status

Risk factors for the development of periodontitis are:

A. Migraine, CHD, diabetes, smoking, childlessness

B. Poor oral hygiene, diabetes, diseases associated with immunodeficiency, menopause

C. Psoriasis, diabetes, smoking, poor oral hygiene

D. All of the named answers apply

Recording patients with an increased risk of complications after oral surgery

A. Is carried out with the involvement of the general practitioner

B. Is usually carried out for all patients at regular intervals by means of medical history questionnaires at the dentist

C. Only carried out by the dentist for patients with known pre-existing conditions

D. If a high-risk patient is identified, the dentist must inform the family doctor of the planned procedure

| 1. **Evaluation** | | | | |
| --- | --- | --- | --- | --- |
|  | 1  Totally agree | 2  Rather agree | 3  Rather disagree | 4  Totally disagree |
| I enjoyed working on the case | 〇 | 〇 | 〇 | 〇 |
| The case inspired me to read more about the connections between dentistry and human medicine on my own. | 〇 | 〇 | 〇 | 〇 |
| My interest in dentistry was aroused. | 〇 | 〇 | 〇 | 〇 |
| The editing had added value for my studies. | 〇 | 〇 | 〇 | 〇 |
| I think that the case should also be applied in subsequent years. | 〇 | 〇 | 〇 | 〇 |
| The processing time was well measured. | 〇 | 〇 | 〇 | 〇 |
| I was able to follow the content well. | 〇 | 〇 | 〇 | 〇 |
| There was a good balance between familiar and new content. | 〇 | 〇 | 〇 | 〇 |
| A common thread was clearly recognizable for me at all times. | 〇 | 〇 | 〇 | 〇 |
| I was able to gain new insights from the case. | 〇 | 〇 | 〇 | 〇 |
| Overall, I was satisfied with the PBL3 case “A case for Erna”. | 〇 | 〇 | 〇 | 〇 |
| I think that PBL is well suited as an interface between human medicine and dentistry in teaching. | 〇 | 〇 | 〇 | 〇 |
| The timing of the course is well chosen for the interface between human medicine and dentistry. | 〇 | 〇 | 〇 | 〇 |
| I found the case to be practically oriented. | 〇 | 〇 | 〇 | 〇 |
| I was taught enough theoretical basics to gain a deeper understanding of the subject matter | 〇 | 〇 | 〇 | 〇 |
| My personal perception of the importance of cooperation between dentists and general practitioners was positively influenced by the case. | 〇 | 〇 | 〇 | 〇 |
| The case was too difficult for me. | 〇 | 〇 | 〇 | 〇 |

**Which main insights do you take away from the case?**

**What did you like in particular?**

**What do you think should be improved?**

1. **Expected answers for knowledge test and risk for disease progression**
2. **Multiple-choice test: 1b, 2a, 3d, 4c, 5b, 6c, 7a, 8a, 9b, 10b.**
3. **Assessment of selected clinical pictures**

| For the following diseases, I estimate the risk of developing a complication (related to the underlying disease) after a dental intervention as follows: | Low | moderate | high |
| --- | --- | --- | --- |
| After Heart valve replacement |  |  |  |
| Diabetes mellitus |  |  |  |
| Pacemaker |  |  |  |
| Renal insufficiency/dialysis |  |  |  |
| Hypertension |  |  |  |
| Glaukoma |  |  |  |
| Rheumatoid arthritis |  |  |  |
| Hip replacement surgery 3 months ago |  |  |  |
| Crohn’s disease |  |  |  |

| I estimate the risk of an increased progression of an oral disease in presence of the selected medical conditions as follows: | Low | moderate | high |
| --- | --- | --- | --- |
| After Heart valve replacement |  |  |  |
| Diabetes mellitus |  |  |  |
| Pacemaker |  |  |  |
| Renal insufficiency/dialysis |  |  |  |
| Hypertension |  |  |  |
| Glaukoma |  |  |  |
| Rheumatoid arthritis |  |  |  |
| Hip replacement surgery 3 months ago |  |  |  |
| Crohn’s disease |  |  |  |
