# Supplementary material for: Raising medical students’ awareness for the interdependence between oral health and systemic diseases - evaluation of a problem-based learning intervention: an exploratory pilot study
Source: BMC Prim Care. 2026 Apr 24;27:161. doi: 10.1186/s12875-026-03323-4 (PMC13109874; doi:10.1186/s12875-026-03323-4)
Supplement: Supplementary file 2 — Supplementary Material 2. [file 12875_2026_3323_MOESM2_ESM.docx]

**Supplement 2: Qualitative Analysis of students´ free text answers**

**Question 2: “What did you like in particular?”**

| **Category title** | **Number of statements n/79 (%)** | **Examples of student responses** |
| --- | --- | --- |
| Inclusion of dentistry | 24 (30.4%) | “Focus on the topic of dental health. I thought it was very good!" |
| Other aspects of content | 18 (22.8%) | “Connection between HbA1c and periodontitis as well as CHD and periodontitis." |
| Design, content, timing | 14 (17.7%) | “The way the case was presented was sympathetic and comprehensible." |
| Practical applicability | 14 (17.7%) | "I think that I will apply what I have learned later on and will also keep an eye on dental health." |
| Gain of knowledge | 4 (5.0%) | „New Insights into the Significance of Dental Disease Patterns for Human Medicine." |
| Selection of additional material | 3 (3.8%) | “The paper was well selected and interesting to read." |
| Holistic approach | 2 (2.5%) | “The ‘holistic’ approach.” |

**Question 3: “What do you think should be improved?”**

| **Category title** | **Number of statements n/108 (%)** | **Examples of student responses** |
| --- | --- | --- |
| Accompanying lecture/seminar | 23 (21.3%) | “Teaching relevant dental diseases as part of a PBL lecture, for example." |
| Unclear content, comprehension problems | 21 (19.4%) | “I would have liked a little more information on the bypass, e.g. why no PCI was performed (...)" |
| Illustrative material/literature references | 17 (15.7%) | “Include images in the processing, films of how the periodontitis is treated." |
| Lack of prior knowledge as an obstacle to discussion and development | 14 (13.1%) | “The lack of basic knowledge often led to collective helplessness.” |
| Problems with data research | 12 (11.1%) | “It is very difficult to find good sources on periodontitis." |
| Time chosen too late in medical studies for integration of  dentistry | 9 (8.3%) | “Relevance of dentistry is addressed too late in the course." |
| Too little content for the allotted time | 9 (8.3%) | “For a PBL case, there was relatively little to work on, we only needed 1 hour on both days.” |
| Other (questionable relevance, problems with questionnaire) | 3 (2.8%) | “I'm just still unsure about the relevance of obtaining a dental status as a GP, as every patient should have a dental check-up every six months anyway.” |
